# Back to the future: the novel art of digital auscultation applied in a prospective observational study of critically ill Covid-19 patients

**DOI:** 10.1186/s41479-024-00131-1

**Published:** 2024-06-05

**Authors:** Evangelos Kaimakamis, Serafeim Kotoulas, Myrto Tzimou, Christos Karachristos, Chrysavgi Giannaki, Vassileios Kilintzis, Leandros Stefanopoulos, Evangelos Chatzis, Nikolaos Beredimas, Bruno Rocha, Diogo Pessoa, Rui Pedro Paiva, Nicos Maglaveras, Militsa Bitzani

**Affiliations:** 1 1st Intensive Care Unit, “G. Papanikolaou” General Hospital, Exochi Thessalonikis, 57010 Greece; 2 Lab of Computing, Medical Informatics and Biomedical Imaging Technologies, The Medical School, Aristotle University, Thessaloniki, Greece; 3 Centre for Informatics and Systems of the University of Coimbra, Department of Informatics Engineering, University of Coimbra, LASI, Coimbra, Portugal; 4 2nd Department of Obstetrics and Gynecology, The Medical School, Thessaloniki, 54124 Greece

**Keywords:** Adventitious lung sounds, ARDS, ICU survival, Lung sounds database, Spectral analysis, Squawks

## Abstract

**Background:**

The Covid-19 pandemic has caused immense pressure on Intensive Care Units (ICU). In patients with severe ARDS due to Covid-19, respiratory mechanics are important for determining the severity of lung damage. Lung auscultation could not be used during the pandemic despite its merit. The main objective of this study was to investigate associations between lung auscultatory sound features and lung mechanical properties, length of stay (LOS) and survival, in adults with severe Covid-19 ARDS.

**Methods:**

Consecutive patients admitted to a large ICU between 2020 and 2021 (*n* = 173) were included. Digital stethoscopes obtained auscultatory sounds and stored them in an on-line database for replay and further processing using advanced AI techniques. Correlation and regression analysis explored relationships between digital auscultation findings and lung mechanics or the ICU outcome. The resulting annotated lung sounds database is also publicly available as supplementary material.

**Results:**

The presence of squawks was associated with the ICU LOS, outcome and 90-day mortality. Other features (age, SOFA score & oxygenation index upon admission, minimum crackle entropy) had significant impact on outcome. Additional features affecting the 90-d survival were age and mean crackle entropy. Multivariate logistic regression showed that survival was affected by age, baseline SOFA, baseline oxygenation index and minimum crackle entropy.

**Conclusions:**

Respiratory mechanics were associated with various adventitious sounds, whereas the lung sound analytics and the presence of certain adventitious sounds correlated with the ICU outcome and the 90-d survival. Spectral features of crackles sounds can serve as prognostic factors for survival, highlighting the importance of digital auscultation.

**Supplementary Information:**

The online version contains supplementary material available at 10.1186/s41479-024-00131-1.

## Background

The Covid-19 pandemic caused by the SARS-CoV2 coronavirus has caused a major pressure on healthcare systems worldwide, with heavy burden especially for Intensive Care Units (ICU), where critically ill patients affected by the disease are hospitalized [1, 2]. These patients express the most severe forms of this multi-systematic disease, with prominent respiratory complications in the form of severe ARDS, dictating the use of invasive mechanical ventilation [3, 4]. Patients with severe ARDS due to Covid-19 seem to entertain a substantially increased risk of death compared to patients with similar symptoms due to H1N1 influenza, after adjusting for SOFA score and other risk factors [4]. This might occur because of the multi-organ insult associated with the disease. The respiratory mechanics of the affected patients play an important role in determining the severity of this insult in the lung parenchyma [5].

Numerous risk factors for adverse prognosis in severely ill patients with Covid-19 have been described in literature. Leoni et al [6] showed that age, obesity, PaO_2_/FiO_2_ ratio, SOFA score and Procalcitonin (PCT) are identifiers of higher ICU mortality. Research conducted by Gallo Marin et al. revealed that on top of the aforementioned parameters, multiple comorbidities, specific CT scan findings, end-organ dysfunction and diverse laboratory abnormalities (increased troponin, lactic dehydrogenase, d-dimer and C-reactive protein) are associated with higher mortality and disease severity [7]. Chen et al. have used metrics from respiratory mechanics as 60-day outcome predictors in ARDS patients utilizing artificial intelligence techniques [8]. Russian researchers have identified the systemic inflammatory response, coagulation disorders and arterial hypertension as risk factors for non-invasive mechanical ventilation or even tracheal intubation in Covid-19 patients [9], whereas, in a large Spanish study from 30 Intensive Care Units during the Covid-19 pandemic [10], the following risk factors led to poor outcomes: Older age, high APACHE II score, acute kidney injury (grades II and III) and presence of septic shock. Overall ICU mortality was reported at 31%. It is evident that ongoing efforts are needed to determine the most appropriate clinical parameters that predict the most adverse outcomes in this pool of patients.

On the other hand, a very practical and useful examination technique, lung auscultation, has not been used during the recent pandemic, due to concerns regarding the examiners’ safety and the burden of personal protective equipment. However, the bulk of information provided by thoracic auscultation is unique and cannot easily be compensated with other means of bedside examination methodologies. This applies for both the determination of the patient disease status and early detection of complications related to invasive mechanical ventilation [11]. Moreover, specific adventitious sounds like squawks, can be indicative of severe complicated pneumonitis or progression to interstitial fibrosis [12], both present in Covid-19 cases. Newer Artificial Intelligence – driven techniques for automatic detection and analysis of lung sounds seem able to provide a more holistic assessment of the respiratory system, enhancing the situational awareness in critically ill respiratory patients [13]. The accuracy of such algorithms is currently limited by the lack of reliable annotated, publicly available databases with auscultation recordings from Covid-19 patients in ICUs. The current databases mainly consist of cough recordings and not adventitious sounds acquisition, although the latter could lead to more robust assessment of the disease status [13], in the context of precision medicine [14, 15].

The aim of this study was to investigate whether there is an association between lung auscultatory sound features as extracted using advanced Machine Learning/ Artificial Intelligence techniques and lung mechanical properties, length of stay and survival, in adult ICU patients, hospitalized due to severe ARDS caused by SARS-CoV2. In addition, the large, unique, comprehensive and annotated lung sounds database from the recorded patients is being made freely available as supplementary material with this paper.

## Methods

The protocol of this study was approved by the ethics committee of “G. Papanikolaou” General Hospital of Thessaloniki, Greece (Reference Number 42/20-05-2020), before the initiation of enrolment and a relative from each participant gave a written informed consent.

Participants were adult intubated ICU patients hospitalized due to SARS-CoV-2. Inclusion criteria were: (1) acute respiratory distress syndrome (ARDS) [3, 16, 17] and (2) positive PCR test for Covid-19 [18]. Exclusion criteria were: (1) age < 18 years and (2) non-requirement for invasive mechanical ventilation upon admission into the ICU. Since this was an original study, there were no previously published data to support a sample size calculation. Thus, all eligible patients who were admitted to the 1st ICU of the above hospital between June 2020 and June 2021 were included. All included patients suffered from severe ARDS according to the Berlin definition and were intubated prior to their entry in the ICU. At the time of the measurements all patients were still at the initial phase of critical illness, fully sedated and under controlled ventilation.

A novel, portable, ear-contact-free digital stethoscope was used to obtain the auscultatory sounds and store them in an on-line database for distant replay and further processing [19]. Respiratory sounds were obtained in auscultatory sessions. Each patient was subjected to an auscultatory session at admission and each time that the treating physician observed a significant alteration in the clinical status thereafter (significant changes in lung mechanical properties, arterial blood gases, ventilation mode, development of a new pulmonary or other infection or significant changes in the bronchial secretions, changes in pulmonary radiological findings, severe hemodynamic instability, multi-organ failure). Six auscultations, lasting 15 s each, were held in every session in pre-specified lung fields -three in each lung- namely the apex front, the base front and the base back [19]. The audio recordings were immediately sent to an Android based tablet PC via Bluetooth connectivity. The CoCross application was used in the Tablet to collect the data, assign them to a predefined patient and securely transmit them to the cloud. Additional clinical and ventilator data could be entered to the application. All input was available to the intensivists via web access in almost real time. Details about the CoCross application and its architecture are provided elsewhere [19].

Two respiratory medicine physicians separately listened to the recordings and characterized them as follows: (1) normal (supplementary audio-file 1), (2) wheezing (audio 2), (3) rhonchi (audio 3), (4) “Velcro” type fine crackles (audio 4), (5) coarse crackles (audio 5), (6) squawks (audio 6), (7) tubular (audio 7), (8) diminished (audio 8), (9) absent (audio 9), (10) pleural rub (audio 10), according to common consensus criteria for classification [12]. In cases of dissension in the original characterization (24.96% of the cases), it was solved by a third respiratory physician who was choosing the most suitable characterization -according to his opinion- between the original two. Since each recording lasted for 15 s, their characterization was done according to the dominant audible adventitious sound, as more than one such sounds could possibly be heard in this time frame. The physicians who characterized the respiratory sounds were blinded to the patients from whom those sounds originated. For each session, the percentage of the recordings which received the same characterization to the total available recordings of that session was calculated. The same calculation of the percentage of the similarly characterized recordings to the total available recordings was also done for each patient separately.

**Fig. 1:**
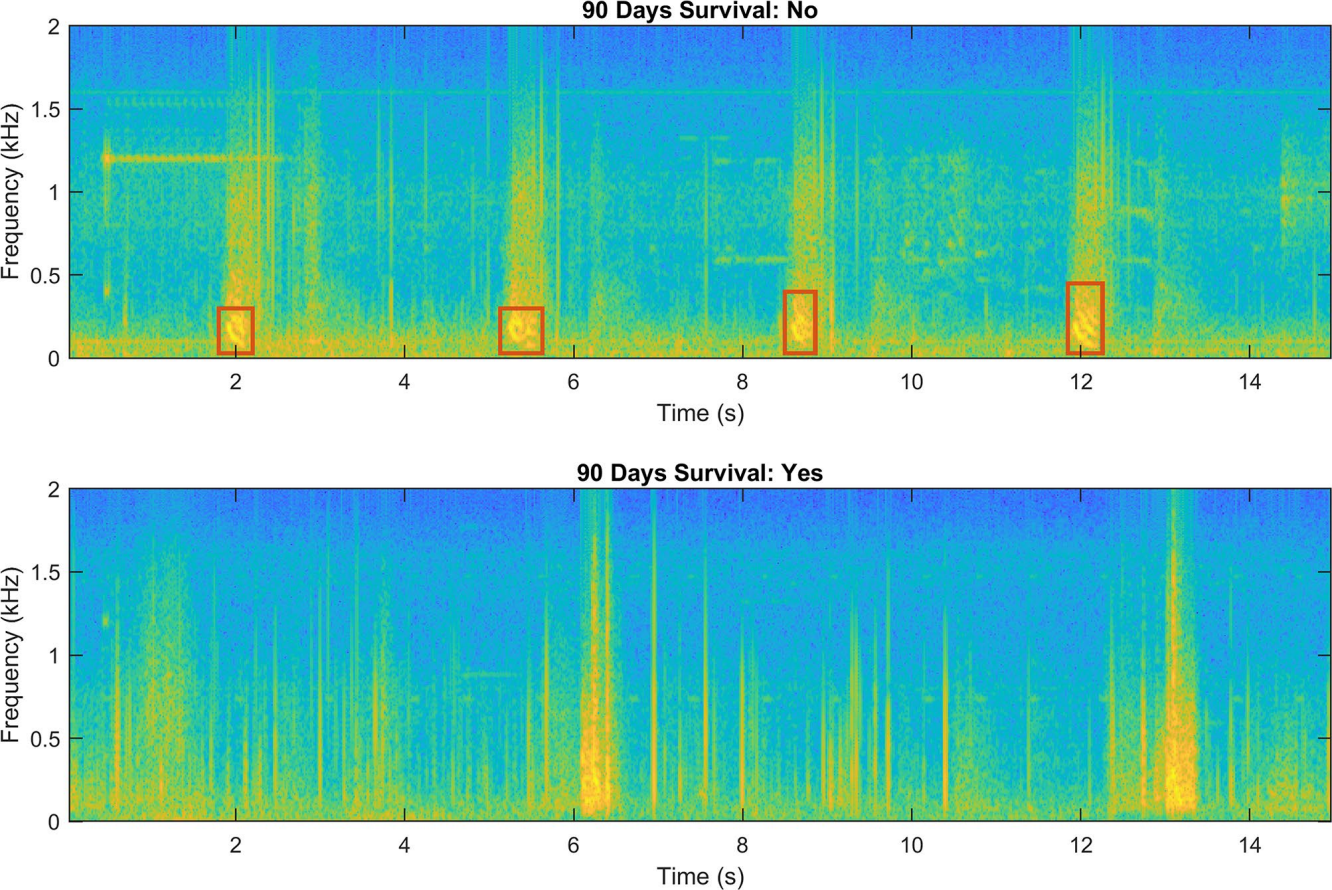
Frequency spectrograms from a patient who survived (below) and from one who perished (above) 90 days after ICU admission. The red squares highlight the presence of squawks as adventitious lung sounds

Furthermore, an audio analysis was performed. The sound analysis method used in this paper was based on the algorithm by Rocha et al [20]. Details on the feature extraction from the audio analysis are provided in a supplementary file. Figure 1 shows characteristic frequency spectrograms from a patient who survived and from one who perished 90 days after ICU admission.

Another investigator, blinded to the sounds of the respiratory auscultations, recorded the characteristics of each patient (age, gender, distinct comorbidities and Charlson comorbidity index [21], acute physiology and chronic health evaluation II [APACHE II] score [22], number of auscultatory sessions per patient, number of distinct respiratory recordings per patient, total days of stay in the ICU, survival and 90-days survival). The conditions under which each auscultatory session took place, namely the patients’ day of stay in the ICU, their daily sequential organ failure assessment (SOFA) score [23], their morning values of hemoglobin, C-reactive protein (CRP) and procalcitonin, as well as the ventilation mode parameters and the arterial blood gas values of the patients at the time of the session, were also recorded, whenever available. From the ventilation mode parameters and the arterial blood gas values, the lung static compliance [5], the lung resistance [5], the oxygenation index [24] and a ventilation equilibrium as the ratio of the minute ventilation (VE) to the partial pressure of carbon dioxide in the arterial blood (PaCO_2_) in milliliters per millimeters of mercury (ml/mmHg) were also recorded or calculated.

Statistical analysis was performed using the SPSS version-20 (IBM-SPSS, Armonk, NY, USA) by a statistician blinded to the research. Continuous variables are presented as mean ± 1 standard deviation (SD) and categorical variables as number/total (%). To investigate correlations between lung mechanical properties (lung static compliance, lung resistance, oxygenation index and ventilation equilibrium) and the percentage of different breath sounds, the parameters of audio analysis, as well as the other quantitative variables of the study, per auscultatory session, a linear regression analysis was performed. The same analysis was performed in order to investigate factors that could correlate with the ICU length of stay for the patients that survived. Factors affecting survival and 90-days survival were also investigated. To perform that, chi-square was used for qualitative variables and independent-samples T-test or Mann Whitney-U test were used for parametric and non-parametric variables, respectively. Normality tests were performed using the Kolmogorov-Smirnov test. The x^2^ test was used for bivariate crosstab associations. Finally, a multivariate logistic regression analysis, using the backward likelihood ratio, was performed between survival and the variables that were statistically significant in the univariate binary logistic regression analysis. The same procedure was followed for 90-day survival. *P* < 0.05 was accepted as statistically significant.

## Results

**Fig. 2:**
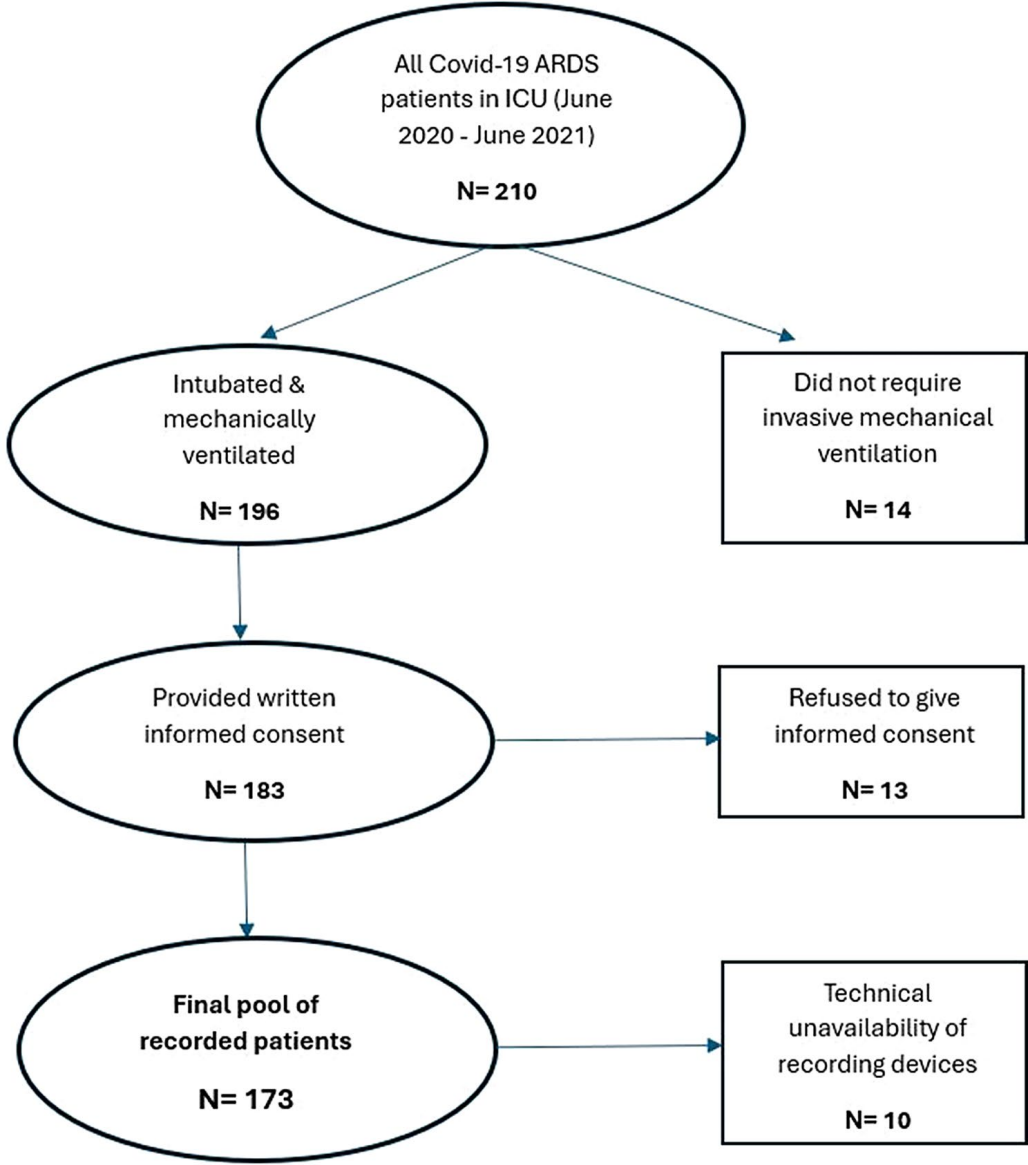
The flowchart for patient selection in the study

In a total of 173 patients, 579 auscultatory sessions were realized. Of the 3474 expected respiratory recordings (579 auscultatory sessions x 6 auscultations per session), 81 (2.33%) were unusable due to technical failures, resulting in 3393 distinct respiratory recordings. A flowchart for patient selection is shown in Fig. 2, whereas Table 1 shows the baseline characteristics of the patients’ cohort as well as the conditions under which auscultatory sessions took place. Supplementary Table 1 shows the characteristics of audio analysis for all the conducted recordings, as well as the mean value of each characteristic per patient.

**Table 1:** Baseline characteristics

Characteristic		Value
Age (years)		65.41 ± 10.16 (Ν = 173)
Gender	female	57/173 (32.9%)
	male	116/173 (67.1%)
Charlson comorbidity index		3.26 ± 1.98 (Ν = 173)
Comorbidities	cardiovascular^1^	126/173 (72.8%)
	metabolic^2^	78/173 (45.1%)
	respiratory^3^	26/173 (15.0%)
	chronic kidney disease	8/173 (4.6%)
	immunosuppression	5/173 (2.9%)
	malignancy	15/173 (8.7%)
	smoking	47/173 (27.2%)
	obesity	42/173 (24.3%)
APACHE II score		16.40 ± 6.39 (Ν = 173)
Daily SOFA score		6.01 ± 2.65 (*N* = 579)
Hemoglobin (g/dl)		10.86 ± 2.00 (*N* = 579)
CRP (mg/dl)		14.02 ± 11.42 (*N* = 413)
PCT (ng/ml)		0.80 ± 2.36 (*N* = 446)
Number of auscultatory sessions per patient		3.35 ± 3.73 (Ν = 173)
Number of auscultations per patient		19.61 ± 20.55 (Ν = 173)
Auscultatory sounds	normal	567/3393 (16.7%)
	wheezing	86/3393 (2.5%)
	rhonchi	481/3393 (14.2%)
	velcro	161/3393 (4.7%)
	crackles	417/3393 (12.3%)
	squawks	352/3393 (10.4%)
	tubular	367/3393 (10.8%)
	diminished	652/3393 (19.2%)
	absence	307/3393 (9.1%)
	pleural rub	3/3393 (0.1%)
Auscultatory sounds per patient (%)	normal	15.92 ± 13.69 (Ν = 173)
	wheezing	1.61 ± 4.79 (Ν = 173)
	rhonchi	14.93 ± 15.77 (Ν = 173)
	velcro	3.74 ± 7.39 (Ν = 173)
	crackles	11.35 ± 12.15 (Ν = 173)
	squawks	8.84 ± 17.26 (Ν = 173)
	tubular	11.69 ± 14.38 (Ν = 173)
	diminished	21.11 ± 18.12 (Ν = 173)
	absence	10.97 ± 16.85 (Ν = 173)
	pleural rub	0.11 ± 1.05 (Ν = 173)
Lung static compliance (ml/cmH_2_O)		44.58 ± 44.99 (*N* = 474)
Lung resistance (cmH_2_O/L/s)		17.98 ± 8.00 (*N* = 215)
Oxygenation index		7.03 ± 4.10 (*N* = 523)
Ventilation equilibrium (ml/mmHg)		227.18 ± 68.01 (*N* = 471)
Survival	Yes	100/173 (57.8%)
	No	73/173 (42.2%)
Days in ICU		21.09 ± 12.59 (*N* = 100)
90-day survival	Yes	92/173 (53.2%)
	No	81/173 (46.8%)

N = number, APACHE II = acute physiology and chronic health evaluation II, SOFA: sequential organ failure assessment, g/dl = gram per deciliter, CRP = c reactive protein, mg/dl = milligram per deciliter, PCT = procalcitonin, ng/ml = nanogram per milliliter, ml/cmH_2_O = milliliter per centimeter of water, cmH_2_O/L/s = centimeter of water per liter per second, ml/mmHg = milliliter per millimeter of mercury, ICU = intensive care unit

^1^Cardiovascular: hypertension, arrythmias, pulmonary emboli, coronary disease, valvular disease, heart failure, peripheral vascular disease

^2^Metabolic: diabetes mellitus, hyperlipidemia, thyroid disease

^3^Respiratory: asthma, chronic obstructive pulmonary disease, pulmonary fibrosis, obstructive sleep apnea hypopnea syndrome

**Fig. 3:**
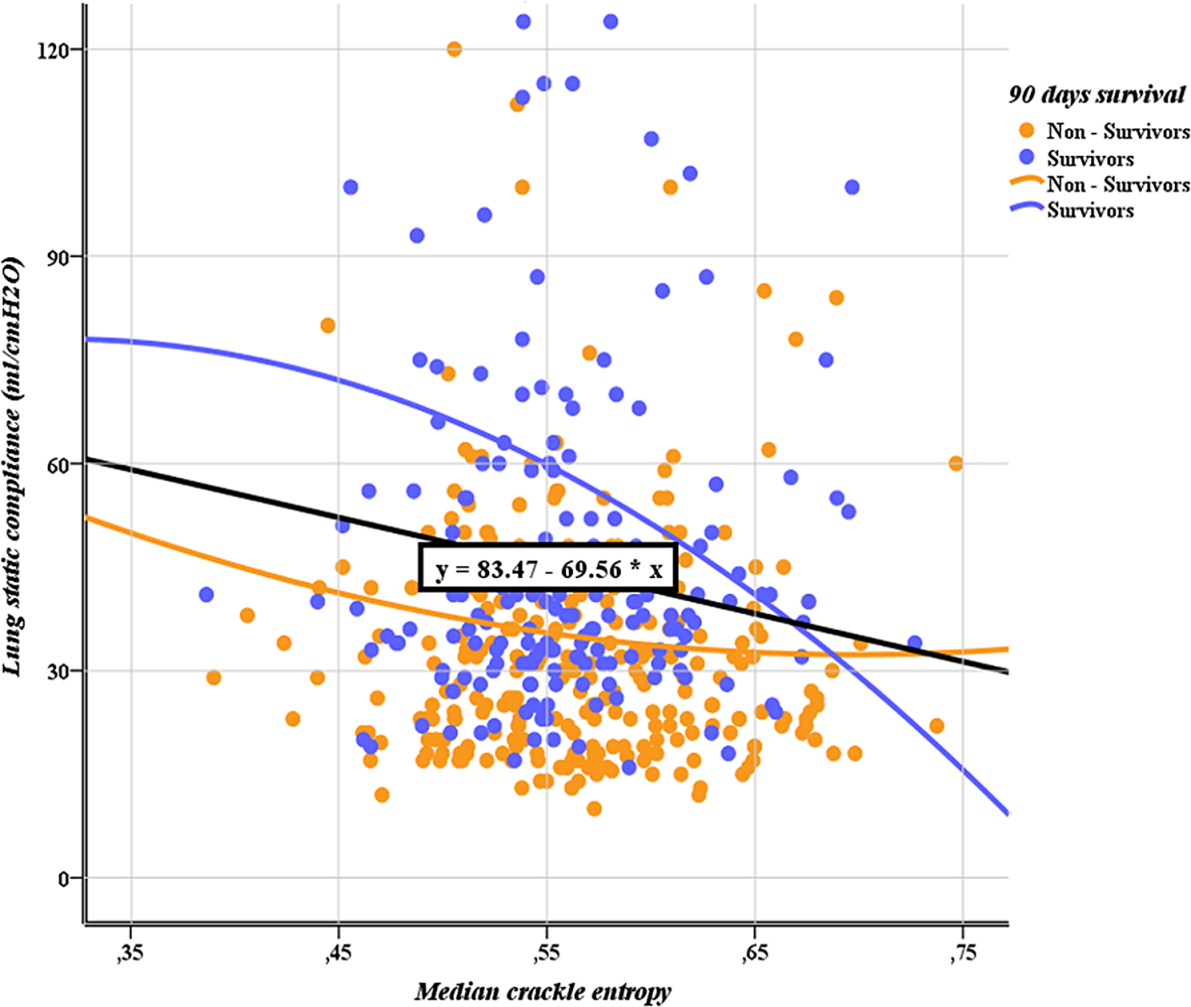
Scatter plot showing the static compliance in relation to median crackle entropy for survivors and non-survivors in the ICU

Lung static compliance was positively correlated with the ICU entry day, the percentage of rhonchi sounds and the percentage of absence of breath sounds, while it was negatively correlated with the daily SOFA score and the percentage of squawks sounds. Lung resistance was positively correlated with the daily SOFA score, the day in ICU, the percentage of wheezing sounds and the squawks sounds, while it was negatively correlated with the percentage of the diminished breath sounds and the percentage of absence of breath sounds. Oxygenation index was positively correlated with the daily SOFA score, CRP, PCT, the percentage of squawks sounds and the percentage of tubular sounds, while it was negatively correlated with the percentage of rhonchi, the percentage of the diminished breath sounds and the percentage of absence of breath sounds. Finally, ventilation equilibrium was positively correlated with the ICU entry day and the percentage of rhonchi. Concerning the associations between the audio analysis parameters and the lung mechanics, static compliance was affected by median and mean crackle entropy, median spectral irregularity, maximum crackle centroid and maximum crackle harmonic ratio. A scatter plot showing the static compliance in relation to median crackle entropy for survivors and non-survivors in the ICU is depicted in Fig. 3. Oxygenation index showed associations with minimum crackle harmonic ratio, minimum spectral brightness ratio, median spectral entropy and irregularity, maximum crackle centroid and maximum crackle harmonic ratio. Resistance was associated with minimum crackle harmonic ratio, minimum spectral brightness ratio, median spectral irregularity, maximum crackle centroid and mean crackle frequency range. Finally, Ventilation Equilibrium was associated with mean crackle duration and median spectral irregularity (more details are shown at Table 2).

**Table 2:** Factors that correlated with lung mechanic properties per auscultatory session

Factor	Lung static compliance		Lung resistance		Oxygenation index		Ventilation equilibrium	
	Standardized coefficients (r)	P (Value)	Standardized coefficients (r)	P (Value)	Standardized coefficients (r)	P (Value)	Standardized coefficients (r)	P (Value)
Daily SOFA score	-0.170	**< 0.001**	0.370	**< 0.001**	0.377	**< 0.001**	-0.060	0.19
CRP (mg/dl)	-0.081	0.13	-0.027	0.73	0.297	**< 0.001**	-0.050	0.35
PCT (ng/ml)	-0.056	0.27	0.023	0.76	0.101	**0.042**	0.019	0.71
Day in ICU	0.117	**0.011**	0.319	**< 0.001**	-0.023	0.60	0.223	**< 0.001**
Auscultatory sound: normal (%)	-0.002	0.97	-0.110	0.11	-0.037	0.40	-0.016	0.73
Auscultatory sound: wheezing (%)	-0.074	0.11	0.178	**0.009**	0.081	0.06	-0.059	0.20
Auscultatory sound: rhonchi (%)	0.137	**0.003**	-0.083	0.22	-0.111	**0.011**	0.107	**0.020**
Auscultatory sound: velcro (%)	0.040	0.39	0.118	0.08	0.080	0.07	-0.019	0.67
Auscultatory sound: crackles (%)	-0.017	0.71	0.017	0.80	0.030	0.49	0.009	0.85
Auscultatory sound: squawks (%)	-0.125	**0.006**	0.337	**< 0.001**	0.233	**< 0.001**	-0.036	0.44
Auscultatory sound: tubular (%)	-0.060	0.19	-0.066	0.34	0.094	**0.032**	0.019	0.67
Auscultatory sound: diminished (%)	0.054	0.24	-0.198	**0.004**	-0.211	**< 0.001**	-0.014	0.76
Auscultatory sound: absence (%)	0.108	**0.018**	-0.198	**0.003**	-0.143	**0.001**	-0.014	0.76
minimum crackle entropy	-0.034	0.47	0.079	0.25	0.018	0.68	0.073	0.11
minimum crackle harmonic ratio	-0.072	0.12	0.164	**0.016**	0.092	**0.036**	0.006	0.89
minimum spectral entropy	0.000	0.99	0.065	0.35	0.074	0.09	0.016	0.73
minimum spectral brightness 800 ratio	0.034	0.46	-0.274	**< 0.001**	-0.104	**0.017**	0.043	0.35
median crackle duration (s)	0.065	0.16	0.061	0.37	0.026	0.56	0.124	**0.007**
median crackle zero-crossing rate	-0.018	0.70	-0.103	0.13	-0.049	0.27	-0.036	0.44
median crackle entropy	-0.107	**0.021**	0.055	0.42	0.079	0.07	-0.008	0.86
median crackle harmonic ratio	0.009	0.85	0.047	0.50	-0.016	0.72	-0.020	0.66
median squawk zero-crossing rate	-0.026	0.57	-0.019	0.78	-0.027	0.55	-0.017	0.71
median spectral entropy	-0.078	0.09	0.119	0.08	0.100	**0.023**	0.017	0.71
median spectral irregularity	0.200	**< 0.001**	-0.238	**< 0.001**	-0.222	**< 0.001**	0.126	**0.006**
maximum crackle centroid (Hz)	0.095	**0.039**	-0.161	**0.018**	-0.201	**< 0.001**	0.012	0.79
maximum crackle harmonic ratio	0.166	**< 0.001**	-0.067	0.33	-0.169	**< 0.001**	0.015	0.75
mean crackle frequency range (Hz)	-0.019	0.68	-0.171	**0.012**	-0.062	0.16	-0.046	0.32
mean crackle entropy	-0.092	**0.046**	0.018	0.80	0.057	0.19	0.038	0.41
mean crackle harmonic ratio	0.046	0.31	0.035	0.61	-0.054	0.22	-0.004	0.92
standard deviation spectral entropy	-0.053	0.25	-0.019	0.78	-0.020	0.65	-0.004	0.93

CI = confidence intervals, SOFA: sequential organ failure assessment, CRP = c reactive protein, PCT = procalcitonin, ICU = intensive care unit. Bold numbers indicate statistically significant correlations

The length of stay in the ICU for the patients who survived was positively associated with the percentage of rhonchi (*r* = 0.225, *p* = 0.024) and the percentage of squawks (*r* = 0.275, *p* = 0.006), while they were negatively associated with the percentage of the diminished breath sounds (*r*= -0.258, *p* = 0.010) and the percentage of absence of breath sounds (*r*= -0.215, *p* = 0.032). In addition, further associations were revealed with median crackle duration (*r*= -0.24, *p* = 0.017) and mean crackle frequency rate (*r*= -0.257, *p* = 0.01) (Supplement Table 2).

Survival was negatively affected by the presence of chronic kidney disease, malignancy, age, Charlson comorbidity index, APACHE II score, baseline SOFA, baseline PCT, baseline oxygenation index and the percentage of squawks. It was also affected by minimum crackle entropy and median squawk zero-crossing rate (Table 3).

**Table 3:** Factors that affected survival

Factor		Non-survivors	Survivors	p (Value)
Gender	female	21/73 (28.8%)	36/100 (36.0%)	0.32
	male	52/73 (71.2%)	64/100 (64.0%)	
Comorbidities	cardiovascular^1^	56/73 (76.7%)	70/100 (70.0%)	0.33
	metabolic^2^	30/73 (41.1%)	48/100 (48.0%)	0.37
	respiratory^3^	11/73 (15.1%)	15/100 (15.0%)	0.99
	chronic kidney disease	7/73 (9.6%)	1/100 (1.0%)	**0.010**
	immunosuppression	4/73 (5.5%)	1/100 (1.0%)	0.16
	malignancy	10/73 (13.7%)	5/100 (5.0%)	**0.045**
	smoking	18/73 (24.7%)	29/100 (29.0%)	0.53
	obesity	18/73 (24.7%)	24/100 (24.0%)	0.92
Age (years)		69.62 ± 8.66	62.33 ± 10.10	**< 0.001**
Charlson comorbidity index		4.07 ± 2.06	2.67 ± 1.70	**< 0.001**
APACHE II score		18.93 ± 6.84	14.55 ± 5.37	**< 0.001**
Baseline SOFA score		6.80 ± 2.16	5.09 ± 2.03	**< 0.001**
Baseline hemoglobin (g/dl)		12.03 ± 1.90	11.85 ± 1.82	0.53
Baseline CRP (mg/dl)		13.63 ± 10.94	13.07 ± 12.14	0.76
Baseline PCT (ng/ml)		1.02 ± 2.57	0.35 ± 1.15	**0.043**
Baseline lung static compliance (ml/cmH_2_O)		35.12 ± 15.19	47.23 ± 49.34	0.05
Baseline lung resistance (cmH_2_O/L/s)		15.33 ± 5.17	15.65 ± 5.08	0.78
Baseline oxygenation index		7.72 ± 3.23	6.33 ± 3.53	**0.011**
Baseline ventilation equilibrium (ml/mmHg)		222.04 ± 59.81	217.37 ± 53.66	0.60
Auscultatory sound: normal (%)		15.89 ± 13.96	15.94 ± 13.56	0.98
Auscultatory sound: wheezing (%)		1.82 ± 4.23	1.45 ± 5.18	0.63
Auscultatory sound: rhonchi (%)		15.15 ± 14.65	14.77 ± 16.62	0.88
Auscultatory sound: velcro (%)		4.59 ± 7.73	3.13 ± 7.10	0.20
Auscultatory sound: crackles (%)		11.51 ± 10.42	11.23 ± 10.33	0.88
Auscultatory sound: squawks (%)		12.39 ± 18.46	6.25 ± 15.93	**0.023**
Auscultatory sound: tubular (%)		14.17 ± 15.16	9.88 ± 13.57	0.05
Auscultatory sound: diminished (%)		18.55 ± 17.93	22.98 ± 18.13	0.11
Auscultatory sound: absence (%)		5.98 ± 11.61	14.61 ± 19.06	**0.001**
minimum crackle entropy		0.35 ± 0.12	0.31 ± 0.14	**0.028**
minimum crackle harmonic ratio		0.13 ± 0.07	0.12 ± 0.09	0.20
minimum spectral entropy		0.74 ± 0.08	0.73 ± 0.14	0.49
minimum spectral brightness 800 ratio		0.20 ± 0.05	0.20 ± 0.05	0.42
median crackle duration (s)		0.03 ± 0.00	0.03 ± 0.01	0.59
median crackle zero-crossing rate		445.69 ± 108.17	446.96 ± 138.54	0.95
median crackle entropy		0.57 ± 0.05	0.55 ± 0.10	0.09
median crackle harmonic ratio		0.36 ± 0.08	0.36 ± 0.09	0.81
median squawk zero-crossing rate		369.17 ± 121.52	327.43 ± 118.50	**0.025**
median spectral entropy		0.81 ± 0.03	0.79 ± 0.12	0.19
median spectral irregularity		0.24 ± 0.06	0.24 ± 0.06	0.31
maximum crackle centroid (Hz)		745.66 ± 158.30	779.26 ± 194.38	0.23
maximum crackle harmonic ratio		0.62 ± 0.11	0.65 ± 0.14	0.14
mean crackle frequency range (Hz)		148.68 ± 60.32	164.61 ± 68.22	0.11
mean crackle entropy		0.56 ± 0.04	0.54 ± 0.09	0.05
mean crackle harmonic ratio		0.36 ± 0.07	0.37 ± 0.09	0.66
standard deviation spectral entropy		0.04 ± 0.03	0.03 ± 0.03	0.41

APACHE II = acute physiology and chronic health evaluation II, SOFA: sequential organ failure assessment, CRP = C-reactive protein, PCT = procalcitonin. Bold numbers indicate statistically significant correlations

^1^Cardiovascular: hypertension, arrythmias, pulmonary emboli, coronary disease, valvular disease, heart failure, peripheral vascular disease

^2^Metabolic: diabetes mellitus, hyperlipidemia, thyroid disease

^3^Respiratory: asthma, chronic obstructive pulmonary disease, pulmonary fibrosis, obstructive sleep apnea hypopnea syndrome

**Fig. 4:**
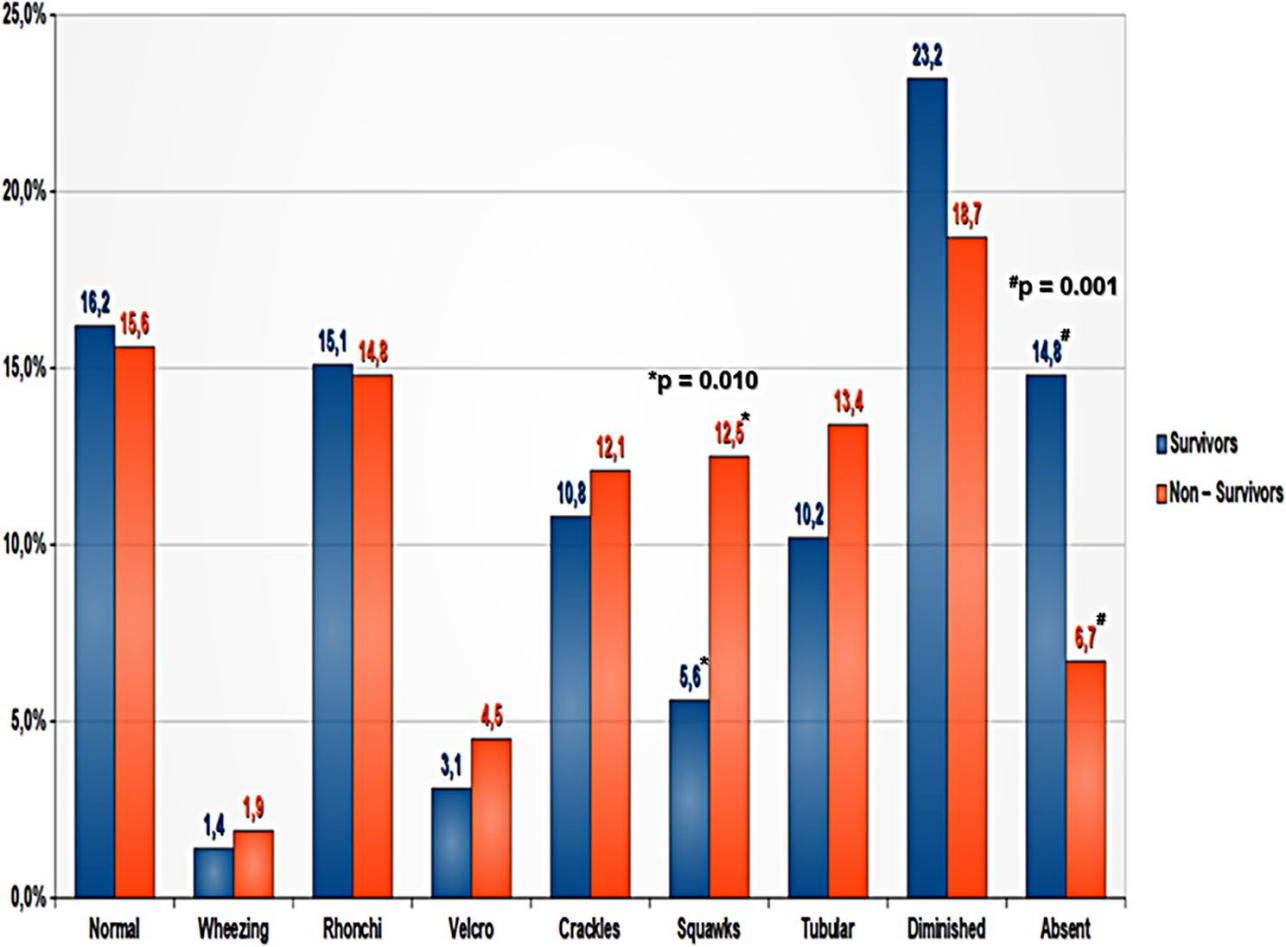
Bar chart with the auscultatory sounds found in patients who survived and those who did not, 90 days later

Ninety-days survival was negatively affected by: the presence of chronic kidney disease, malignancy, age, Charlson comorbidity index, APACHE II score, baseline SOFA, baseline PCT, baseline oxygenation index and the percentage of squawks. Moreover, it was affected by median and mean crackle entropy, as well as median squawk zero-crossing rate (Table 4). Figure 4 shows a bar chart with the auscultatory sounds found in patients who survived and those who did not 90 days later (Fig. 3).

**Table 4:** Factors that affected 90-days survival

Factor		Non-survivors	Survivors	p (Value)
Gender	female	24/81 (29.6%)	33/92 (35.9%)	0.38
	male	57/81 (70.4%)	59/92 (64.1%)	
Comorbidities	cardiovascular^1^	62/81 (76.5%)	64/92 (69.6%)	0.30
	metabolic^2^	38/81 (46.9%)	40/92 (43.5%)	0.65
	respiratory^3^	14/81 (17.3%)	12/92 (13.0%)	0.44
	chronic kidney disease	7/81 (8.6%)	1/92 (1.1%)	**0.026**
	immunosuppression	4/81 (4.9%)	1/92 (1.1%)	0.19
	malignancy	12/81 (14.8%)	3/92 (3.3%)	**0.007**
	smoking	21/81 (25.9%)	26/92 (28.3%)	0.73
	obesity	22/81 (27.2%)	20/92 (21.7%)	0.41
Age (years)		69.52 ± 8.37	61.78 ± 10.25	**< 0.001**
Charlson comorbidity index		4.16 ± 2.17	2.47 ± 1.39	**< 0.001**
APACHE II score		19.12 ± 6.90	14.00 ± 4.79	**< 0.001**
Baseline SOFA score		6.78 ± 2.12	4.96 ± 2.01	**< 0.001**
Baseline hemoglobin (g/dl)		12.02 ± 1.89	11.85 ± 1.82	0.55
Baseline CRP (mg/dl)		13.29 ± 10.74	13.32 ± 12.40	0.99
Baseline PCT (ng/ml)		1.10 ± 2.72	0.23 ± 0.34	**0.006**
Baseline lung static compliance (ml/cmH_2_O)		35.70 ± 15.67	47.72 ± 51.08	0.05
Baseline lung resistance (cmH_2_O/L/s)		15.32 ± 5.36	15.67 ± 5.08	0.75
Baseline oxygenation index		7.56 ± 3.22	6.34 ± 3.59	**0.025**
Baseline ventilation equilibrium (ml/mmHg)		221.30 ± 57.54	217.67 ± 55.44	0.68
Auscultatory sound: normal (%)		15.60 ± 13.65	16.20 ± 13.79	0.78
Auscultatory sound: wheezing (%)		1.86 ± 4.13	1.39 ± 5.32	0.52
Auscultatory sound: rhonchi (%)		14.81 ± 14.41	15.04 ± 16.96	0.93
Auscultatory sound: velcro (%)		4.49 ± 7.46	3.08 ± 7.30	0.21
Auscultatory sound: crackles (%)		12.03 ± 10.60	10.75 ± 13.40	0.49
Auscultatory sound: squawks (%)		12.49 ± 18.65	5.63 ± 15.34	**0.010**
Auscultatory sound: tubular (%)		13.36 ± 14.71	10.21 ± 13.99	0.15
Auscultatory sound: diminished (%)		18.74 ± 17.74	23.19 ± 18.30	0.11
Auscultatory sound: absence (%)		6.65 ± 13.04	14.76 ± 18.87	**0.001**
minimum crackle entropy		0.35 ± 0.12	0.31 ± 0.14	0.06
minimum crackle harmonic ratio		0.13 ± 0.07	0.12 ± 0.09	0.24
minimum spectral entropy		0.74 ± 0.08	0.73 ± 0.14	0.46
minimum spectral brightness 800 ratio		0.20 ± 0.05	0.20 ± 0.05	0.59
median crackle duration (s)		0.03 ± 0.00	0.03 ± 0.01	0.76
median crackle zero-crossing rate		450.42 ± 111.81	442.87 ± 138.28	0.70
median crackle entropy		0.57 ± 0.05	0.54 ± 0.10	**0.046**
median crackle harmonic ratio		0.36 ± 0.07	0.36 ± 0.10	0.92
median squawk zero-crossing rate		372.17 ± 119.65	321.10 ± 118.11	**0.005**
median spectral entropy		0.81 ± 0.03	0.78 ± 0.13	0.09
median spectral irregularity		0.24 ± 0.06	0.24 ± 0.06	0.35
maximum crackle centroid (Hz)		754.39 ± 158.10	774.44 ± 198.26	0.47
maximum crackle harmonic ratio		0.62 ± 0.11	0.65 ± 0.14	0.15
mean crackle frequency range (Hz)		149.67 ± 60.43	165.13 ± 68.83	0.12
mean crackle entropy		0.56 ± 0.04	0.54 ± 0.10	**0.038**
mean crackle harmonic ratio		0.36 ± 0.06	0.37 ± 0.09	0.70
standard deviation spectral entropy		0.04 ± 0.03	0.03 ± 0.03	0.19

APACHE II = acute physiology and chronic health evaluation II, SOFA: sequential organ failure assessment, CRP = C-reactive protein, PCT = procalcitonin. Bold numbers indicate statistically significant correlations

^1^Cardiovascular: hypertension, arrythmias, pulmonary emboli, coronary disease, valvular disease, heart failure, peripheral vascular disease

^2^Metabolic: diabetes mellitus, hyperlipidemia, thyroid disease

^3^Respiratory: asthma, chronic obstructive pulmonary disease, pulmonary fibrosis, obstructive sleep apnea hypopnea syndrome

Bivariate analysis for associations between presence of certain adventitious sounds and the outcome were performed with the x^2^ test. They revealed that ICU survival was associated with the presence of squawks (x^2^ = 8.4, *p* = 0.004), whereas 90d survival was associated with both the presence of squawks (x^2^ = 10, *p* = 0.002) and “Velcro” crackles (x^2^ = 4.18, *p* = 0.048).

Multivariate logistic regression analysis showed that for every year of increase in age, the odds ratio of death was also increased by 1.077, *p* = 0.001. The same was applied for every unit of increase in the baseline SOFA score (OR = 1.328, *p* = 0.005) for every unit of increase in the baseline oxygenation index (OR = 1.140, *p* = 0.033) and for every unit of increase of minimum crackle entropy in the audio analysis (OR = 39.974, *p* = 0.024). Finally, for every unit of increase in the percentage of squawks breath sounds the odds ratio of death in 90 days was increased by 1.025, *p* = 0.034 (Table 5).

**Table 5:** Multivariate binary logistic regression analysis between survival and 90-days survival and the factors that were significant in the univariate analysis

Survival		
Factor	OR (95% CI)	P (Value)
Age (years)	1.077 (1.032–1.124)	**0.001**
Heart failure (no vs. yes)	2.813 (0.387–20.442)	0.31
Chronic kidney disease (no vs. yes)	3.376 (0.335–34.045)	0.30
Malignancy (no vs. yes)	1.355 (0.246–7.473)	0.73
Charlson comorbidity index	1.104 (0.843–1.445)	0.47
APACHE II	0.978 (0.891–1.074)	0.65
Baseline SOFA score	1.328 (1.090–1.620)	**0.005**
Baseline PCT (ng/ml)	1.066 (0.816–1.392)	0.64
Baseline oxygenation index	1.140 (1.011–1.286)	**0.033**
Auscultatory sound: squawks (%)	1.020 (1.000–1.040)	**0.05**
minimum crackle entropy	39.974 (1.637–976.366)	**0.024**
median squawk zero-crossing rate	1.001 (0.997–1.005)	0.64
**90-days survival**		
**Factor**	**OR (95% CI)**	**P (Value)**
Age (years)	1.053 (0.999–1.110)	**0.05**
Heart failure (no vs. yes)	6.732 (0.556–81.509)	0.13
Chronic kidney disease (no vs. yes)	0.782 (0.054–11.422)	0.86
Malignancy (no vs. yes)	1.187 (0.177–7.943)	0.86
Charlson comorbidity index	1.270 (0.940–1.717)	0.12
APACHE II	0.989 (0.896–1.092)	0.83
Baseline SOFA score	1.240 (0.996–1.544)	0.06
Baseline PCT (ng/ml)	1.838 (0.849–3.976)	0.12
Baseline oxygenation index	1.126 (0.995–1.274)	0.06
Auscultatory sound: squawks (%)	1.025 (1.002–1.049)	**0.034**
median crackle entropy	0.172 (0.000–1532353.917)	0.83
median squawk zero-crossing rate	1.002 (0.998–1.006)	0.25
mean crackle entropy	0.939 (44722.225–2,129,163,370)	**0.05**

OR = odds ratio, CI = confidence intervals, vs. = versus, APACHE II = acute physiology and chronic health evaluation II, SOFA: sequential organ failure assessment, PCT = procalcitonin

The database derived by this study with the obtained respiratory sound recordings and X-ray images can be found in the following link to a public repository https://figshare.com/s/e5af036d5ca46150eac4. The data is organized by subject.

## Discussion

In this paper, a novel method of obtaining and analyzing auscultation sounds from critically ill Covid-19 patients was presented. The new methodology involves digital recording of lung sounds and proper utilization of auditory analysis algorithms in order to retrieve diagnostic indices from severely affected patients. In the study population, respiratory mechanics presented strong associations with various adventitious sounds, whereas the lung sound analytics and the presence of certain adventitious sounds were well associated with the ICU survival and the 90-days survival. To the best of our knowledge, this is the first effort correlating specific elements from lung auscultation with the outcome in critically ill Covid-19 patients. It is also accompanied by the first publicly available dataset with lung sounds from severely ill Covid-19 patients in an ICU environment. The value of such unique and new databases is profound, since they can help researchers to refine automated analysis of the condition of the lung mechanics and provide better support and educational material to the clinicians in complex situations. This concept is in alignment with the notion of personalized medicine, which is in turn considered crucial for better management of critically ill patients in many disease patterns and especially Covid-19 [14]. So far, researchers have delivered expert systems for diagnosis of Covid-19 based on remote breath and cough analysis [25, 26], but none of them was focused on critically ill patients under mechanical ventilation. Optimal computational analysis of lung sounds using tools like convolutional neural networks or support vector machines, may lead to more precise diagnoses and assessments of lung mechanics [12, 27]. In the near future, utilization of supplementary point-of-care tools like Electrical Impedance Tomography (EIT) and lung ultrasound, coupled with other assessments of macro-respiratory mechanics and regional lung expansion (like the ones provided by lung auscultation) can offer personalized management of severe ARDS [28, 29]. It is also mentioned that protective lung ventilation in severe ARDS can be achieved through closed-loop ventilator modes assisted by lung sounds [30], another proof that lung auscultation still plays an important role in clinical management of critically ill patients in the ICU.

Regarding other noteworthy findings, the presence of squawks was associated with the ICU length of stay, but also with the ICU and the 28-day mortality. This signifies the important role of lung auscultation in assessing the severity of the lung insult and necessitates the recognition of specific adventitious sounds that may indicate unfavorable progression of the disease. It seems that squawks are clear indicators of severe lung damage and/or initiation of the fibrotic state of severe ARDS, either as a process through the disease pathway [31] or as a consequence of mechanical ventilation [32]. Diminished or absent lung sounds were also helpful in determining the most severe cases of lung insult in our study. This is important, since early identification of respiratory complications is essential in order to modify the ventilation settings and avoid irreversible lung damage, in view of the fact that Covid-19 patients who had milder lung complications had better survival rates and satisfactory quality of life one year after discharge from the ICU [27]. In this context, proper identification of the key prognostic factors for better ICU outcome is paramount, as it will allow the design of interventions to improve outcomes and effectively allocate the scarce resources [7].

Apart from the presence of squawks, other features (namely age, the initial SOFA score, the oxygenation index upon ICU admission and the minimum crackle entropy) had a significant impact on ICU survival. The additional features that affected the 90-d survival were age and the mean crackle entropy, as revealed by the multivariate logistic regression. Interestingly, specific spectral features of the crackles adventitious sounds can serve as prognostic factors for survival, highlighting the importance of proper auscultation in these patients. Concerning the rest of the parameters that affected survival, our study is in agreement with other researchers who have revealed that age, SOFA, oxygenation index did play a role in predicting the ICU survival [6, 7]. These researchers have also shown that comorbid conditions and PCT affect survival, features that were also found to correlate with survival in our cohort, although they did not seem to reach adequate significance level in multivariate analysis.

It is worth noticing that 90-days survival was only affected by age and certain adventitious sounds, which deems necessary to further explore the association of such auscultation findings with the underlying respiratory mechanics in ARDS patients. In our study, spectral sound features were associated with respiratory mechanics, though the latter did not correlate well with survival, a finding which is in disagreement with some published studies [8]. Statistically significant associations were found between various spectral sound features and static respiratory compliance and resistance. These associations included both the oxygen and the carbon dioxide exchange, as depicted by correlations with oxygenation index and ventilation equilibrium. Despite the lack of direct link between conventionally measured respiratory dynamics and the outcome in severely ill patients, such connections are found between the outcome and various auscultation sounds and parameters. This is an indication of the importance of auscultatory findings for proper assessment of the respiratory system in critically ill patients and rings a bell for the tendency to refrain from this traditional form of clinical examination in favor of “contemporary” examination modalities. The art of precise auscultation can now be aided by technology to allow performing of this important intervention even in restricted environments and moreover, expand its potential by automated extraction of extra features from lung sounds, like spectral parameters and more. Also, alternative models of repetitive data interpretation, like General Linear Models (GLM) could facilitate deeper insight into the desired associations.

Among the weaknesses of the study is its monocentric nature and the fact that there was a small number of patients with only one sound recording conducted, a fact that may limit its capability to correctly associate all the sound features with the lung mechanics and the measured outcomes. In addition, due to the observational nature of the study, sample size calculation was not performed. This is considered an exploratory analysis; thus, its conclusions are of relatively limited power and require further experiments in larger sample sizes.

On the other hand, another aspect of chest sounds recording is the simultaneous recording of heart sounds and murmurs which could give valuable insight into the heart complications during the Covid-19 pandemic. The disease is known to affect the heart and occasionally lead to myocardial injury that may have various clinical presentations, it is overall associated with high rates of complications and mortality and possible long-term cardiac impairments in survivors. Both pathophysiological mechanisms and long-term evolution of survivors still deserve further investigations with solutions like CoCross [33]. Further analysis of the obtained heart sounds from our study is ongoing.

For the future, the CoCross application could be utilized to perform a clinical clustering of Covid-19 cases for optimal monitoring and management of Post-Acute Covid-19 Syndrome (PACS), which is very common in patients hospitalized in Intensive Care settings because of the disease. Ravaglia et al. have proposed three distinct clusters of such patients, namely chronic fibrosing, those with acute/subacute lung injury and those with vascular changes [34]. The description of these clusters implies that there will be unique auscultation findings in each of these groups, enabling applications like CoCross to effectively categorize the patients and provide targeted monitoring solutions. Other researchers have confirmed that more than one-third of patients present persistent interstitial lung abnormalities 2 years after Covid-19 infection, which are associated with respiratory symptoms [34]. These cases could benefit from remote monitoring applications utilizing lung auscultation and automated analysis techniques for more effective and meaningful clinical assessment.

## Conclusion

The type and spectral features of adventitious lung sounds can serve as prognostic factors for survival in Covid-19 ARDS. Digital auscultation has proved to be feasible & beneficial in critically ill patients with ARDS, even in confined conditions with extra protective equipment. This study was the first effort which successfully associated specific traits from lung auscultation with the outcome in critically ill Covid-19 patients. It is also accompanied by the first publicly available dataset with lung sounds from severely ill Covid-19 patients in an ICU environment.

### Electronic supplementary material

Below is the link to the electronic supplementary material.

Supplementary Material 1

Supplementary Material 2

Supplementary Material 3

Supplementary Material 4

Supplementary Material 5

Supplementary Material 6

Supplementary Material 7

Supplementary Material 8

Supplementary Material 9

Supplementary Material 10

Supplementary Material 11

Supplementary Material 12

## Data Availability

The database derived by this study with the obtained respiratory sound recordings and X-ray images can be found in the following link to a public repository https://figshare.com/s/e5af036d5ca46150eac4. The data is organized by subject.

## Abbreviations

ICU: Intensive Care Units
LOS: length of stay
PCT: Procalcitonin
CT: Computed Tomography
ARDS: Acute Respiratory Distress Syndrome
AI: Artificial Intelligence
PCR: Polymerase Chain Reaction
APACHE II: Acute Physiology and Chronic Health Evaluation II
SOFA: Sequential Organ Failure Assessment
CRP: C-Reactive Protein
VE: Minute Ventilation
PaCO_2_: Partial Pressure of Carbon Dioxide in the Arterial Blood
EIT: Electrical Impedance Tomography
PACS: Post-Acute Covid-19 Syndrome
GLM: General Linear Models

## References

[1] Armstrong RA, Kane AD, Kursumovic E, Oglesby FC, Cook TM (2021). Mortality in patients admitted to intensive care with COVID-19: an updated systematic review and meta-analysis of observational studies. Anaesthesia.

[2] Serafim RB, Povoa P, Souza-Dantas V, Kalil AC, Salluh JIF (2021). Clinical course and outcomes of critically ill patients with COVID-19 infection: a systematic review. Clin Microbiol Infect.

[3] Bernard GR, Artigas A, Brigham KL, Carlet J, Falke K, Hudson L, Lamy M, LeGall JR, Morris A, Spragg R. Report of the American-European consensus conference on ARDS: definitions, mechanisms, relevant outcomes and clinical trial coordination. The Consensus Committee. *Intensive Care Med* 1994, 20(3):225–232. 10.1007/BF01704707 8014293

[4] Hernandez-Cardenas C, Lugo-Goytia G, Hernandez-Garcia D, Perez-Padilla R (2022). Comparison of the clinical characteristics and mortality in acute respiratory distress syndrome due to COVID-19 versus due to Influenza A-H1N1pdm09. Med Intensiva (Engl Ed).

[5] Hess DR (2014). Respiratory mechanics in mechanically ventilated patients. Respir Care.

[6] Leoni MLG, Lombardelli L, Colombi D, Bignami EG, Pergolotti B, Repetti F, Villani M, Bellini V, Rossi T, Halasz G (2021). Prediction of 28-day mortality in critically ill patients with COVID-19: development and internal validation of a clinical prediction model. PLoS ONE.

[7] Gallo Marin B, Aghagoli G, Lavine K, Yang L, Siff EJ, Chiang SS, Salazar-Mather TP, Dumenco L, Savaria MC, Aung SN (2021). Predictors of COVID-19 severity: a literature review. Rev Med Virol.

[8] Chen L, Grieco DL, Beloncle F, Chen GQ, Tiribelli N, Madotto F, Fredes S, Lu C, Antonelli M, Mercat A (2022). Partition of respiratory mechanics in patients with acute respiratory distress syndrome and association with outcome: a multicentre clinical study. Intensive Care Med.

[9] Chebotareva N, Berns S, Androsova T, Moiseev S (2022). Risk factors for invasive and non-invasive ventilatory support and mortality in hospitalized patients with COVID-19. Med Intensiva (Engl Ed).

[10] Ferrando C, Mellado-Artigas R, Gea A, Arruti E, Aldecoa C, Bordell A, Adalia R, Zattera L, Ramasco F, Monedero P (2020). Patient characteristics, clinical course and factors associated to ICU mortality in critically ill patients infected with SARS-CoV-2 in Spain: a prospective, cohort, multicentre study. Rev Esp Anestesiol Reanim (Engl Ed).

[11] Zhu J, Tan Y, Huang B, Zhu Y, Gao XH (2021). Don’t throw the stethoscope away!. Eur Heart J.

[12] Bohadana A, Izbicki G, Kraman SS (2014). Fundamentals of lung auscultation. N Engl J Med.

[13] Xia T, Han J, Mascolo C (2022). Exploring machine learning for audio-based respiratory condition screening: a concise review of databases, methods, and open issues. Exp Biol Med (Maywood).

[14] Annane D, Meduri GU (2022). Precision medicine for corticotherapy in COVID-19. Intensive Care Med.

[15] Witzenrath M, Welte T. A leap towards personalised therapy of acute lung injury. Eur Respir J 2022, 60(6). 10.1183/13993003.01808-2022 36522140

[16] Force ADT, Ranieri VM, Rubenfeld GD, Thompson BT, Ferguson ND, Caldwell E, Fan E, Camporota L, Slutsky AS (2012). Acute respiratory distress syndrome: the Berlin definition. JAMA.

[17] Grasselli G, Calfee CS, Camporota L, Poole D, Amato MBP, Antonelli M, Arabi YM, Baroncelli F, Beitler JR, Bellani G et al. ESICM guidelines on acute respiratory distress syndrome: definition, phenotyping and respiratory support strategies. Intensive Care Med 2023. 10.1007/s00134-023-07050-7 PMC10354163 37326646

[18] Corman VM, Landt O, Kaiser M, Molenkamp R, Meijer A, Chu DK, Bleicker T, Brunink S, Schneider J, Schmidt ML et al. Detection of 2019 novel coronavirus (2019-nCoV) by real-time RT-PCR. Euro Surveill 2020, 25(3). 10.2807/1560-7917.ES.2020.25.3.2000045 PMC6988269 31992387

[19] Kilintzis V, Beredimas N, Kaimakamis E, Stefanopoulos L, Chatzis E, Jahaj E, Bitzani M, Kotanidou A, Katsaggelos AK, Maglaveras N. CoCross: an ICT platform enabling Monitoring Recording and Fusion of clinical information chest sounds and imaging of COVID-19 ICU patients. Healthc (Basel) 2022, 10(2). 10.3390/healthcare10020276 PMC8871733 35206889

[20] Rocha BM, Pessoa D, Cheimariotis GA, Kaimakamis E, Kotoulas SC, Tzimou M, Maglaveras N, Marques A, de Carvalho P, Paiva RP (2021). Detection of squawks in respiratory sounds of mechanically ventilated COVID-19 patients. Annu Int Conf IEEE Eng Med Biol Soc.

[21] Charlson ME, Pompei P, Ales KL, MacKenzie CR (1987). A new method of classifying prognostic comorbidity in longitudinal studies: development and validation. J Chronic Dis.

[22] Knaus WA, Draper EA, Wagner DP, Zimmerman JE (1985). APACHE II: a severity of disease classification system. Crit Care Med.

[23] Vincent JL, Moreno R, Takala J, Willatts S, De Mendonca A, Bruining H, Reinhart CK, Suter PM, Thijs LG (1996). The SOFA (Sepsis-related Organ failure Assessment) score to describe organ dysfunction/failure. On behalf of the Working Group on Sepsis-related problems of the European Society of Intensive Care Medicine. Intensive Care Med.

[24] Trachsel D, McCrindle BW, Nakagawa S, Bohn D (2005). Oxygenation index predicts outcome in children with acute hypoxemic respiratory failure. Am J Respir Crit Care Med.

[25] Kontou P, Kotoulas SC, Kalliontzis S, Synodinos-Kamilos S, Akritidou S, Kaimakamis E, Anisoglou S, Manika K (2023). Evaluation of Pain scales and Outcome in critically ill patients of a Greek ICU. J Pain Palliat Care Pharmacother.

[26] Athanasiou N, Baou K, Papandreou E, Varsou G, Amfilochiou A, Kontou E, Pataka A, Porpodis K, Tsiouprou I, Kaimakamis E (2023). Association of sleep duration and quality with immunological response after vaccination against severe acute respiratory syndrome coronavirus-2 infection. J Sleep Res.

[27] Lavrentieva A, Kaimakamis E, Voutsas V, Bitzani M (2023). An observational study on factors associated with ICU mortality in Covid-19 patients and critical review of the literature. Sci Rep.

[28] Bitker L, Talmor D, Richard JC. Imaging the acute respiratory distress syndrome: past, present and future. Intensive Care Med 2022. 10.1007/s00134-022-06809-8 PMC9281340 35833958

[29] Wu Y, Rocha BM, Kaimakamis E, Cheimariotis G-A, Petmezas G, Chatzis E, Kilintzis V, Stefanopoulos L, Pessoa D, Marques A et al. A deep learning method for predicting the COVID-19 ICU patient outcome fusing X-rays, respiratory sounds, and ICU parameters. Expert Syst Appl 2024, 235.

[30] Buiteman-Kruizinga LA, Serpa Neto A, Schultz MJ (2022). Automation to improve lung protection. Intensive Care Med.

[31] Pessoa D, Rocha BM, Strodthoff C, Gomes M, Rodrigues G, Petmezas G, Cheimariotis GA, Kilintzis V, Kaimakamis E, Maglaveras N et al. BRACETS: Bimodal repository of auscultation coupled with electrical impedance thoracic signals. *Comput Methods Programs Biomed* 2023, 240:107720. 10.1016/j.cmpb.2023.107720 37544061

[32] Tsiftsoglou SA, Gavriilaki E, Touloumenidou T, Koravou EE, Koutra M, Papayanni PG, Karali V, Papalexandri A, Varelas C, Chatzopoulou F (2023). Targeted genotyping of COVID-19 patients reveals a signature of complement C3 and factor B coding SNPs associated with severe infection. Immunobiology.

[33] Helms J, Combes A, Aissaoui N (2022). Cardiac injury in COVID-19. Intensive Care Med.

[34] Han X, Chen L, Fan Y, Alwalid O, Jia X, Zheng Y, Liu J, Li Y, Cao Y, Gu J (2023). Longitudinal Assessment of chest CT findings and pulmonary function after COVID-19 infection. Radiology.

