# Supplementary material for: Back to the future: the novel art of digital auscultation applied in a prospective observational study of critically ill Covid-19 patients
Source: Pneumonia (Nathan). 2024 Jun 5;16:9. doi: 10.1186/s41479-024-00131-1 (PMC11151547; doi:10.1186/s41479-024-00131-1)
Supplement: Supplementary file 1 — Supplementary Material 1 [file 41479_2024_131_MOESM1_ESM.docx]

**SUPPLEMENTARY MATERIAL 1**

**Article Tile: “Back to the future: The novel art of digital auscultation applied in critically ill Covid-19 patients”**

**METHODS SECTION: DETAILS ON FEATURE EXTRACTION FROM AUDIO ANALYSIS**

An audio analysis was performed based on the signals obtained by the CoCross application from the critically ill Covid-19 patients in the ICU environment. The sound analysis method used in this paper was based on the algorithm by Rocha et al. For each day containing several recordings, we identified possible adventitious respiratory sounds, clustered according to duration into potential wheezes, crackles, and squawks. For each type of sound, the component with duration closest to the median duration of the cluster was chosen as the candidate component, and 69 features were extracted for each candidate, including graphical features, spectral features, Mel-frequency cepstral coefficients and gammatone cepstral coefficients. On a separate path, 81 spectral features, melodic features, and MFCCs were extracted for each day of recordings. A window of 64 ms was used to extract the spectrograms from which the features were extracted. In total, 288 features were extracted. Then, we computed statistical summaries of those features, namely minimum, median, maximum, mean, and standard deviation, reaching a total of 1440 features. Of those, the following were the most relevant: minimum crackle entropy, minimum crackle harmonic ratio, minimum spectral entropy, minimum spectral brightness 800 ratio, median crackle duration, median crackle zero-crossing rate, median crackle entropy, median crackle harmonic ratio, median squawk zero-crossing rate, median spectral entropy, median spectral irregularity, maximum crackle centroid, maximum crackle harmonic ratio, mean crackle frequency range, mean crackle entropy, mean crackle harmonic ratio and standard deviation spectral entropy. Spectral entropy, the most relevant feature in this analysis, is a measure of the complexity of the spectrum. Another relevant feature is the harmonic ratio, which is computed as the maximum of the normalized autocorrelation. Regarding the other relevant features, spectral brightness 800 ratio is the ratio between spectral brightness at 800 and 100 Hz, median crackle duration is the median of the duration of the candidate crackles, zero-crossing rate is the waveform sign-change rate, spectral irregularity is an estimation of the spectral peaks’ variability, spectral centroid is the center of mass of the spectral distribution, and mean crackle frequency range is the mean range of frequencies of candidate crackles. The mean value of each parameter per patient was also calculated.
