# Supplementary material for: Back to the future: the novel art of digital auscultation applied in a prospective observational study of critically ill Covid-19 patients
Source: Pneumonia (Nathan). 2024 Jun 5;16:9. doi: 10.1186/s41479-024-00131-1 (PMC11151547; doi:10.1186/s41479-024-00131-1)
Supplement: Supplementary file 2 — Supplementary Material 2 [file 41479_2024_131_MOESM2_ESM.docx]

**SUPPLEMENTARY MATERIAL 2**

**Article Tile: “Back to the future: The novel art of digital auscultation applied in critically ill Covid-19 patients”**

**SUPPLEMENT TABLES**

Supplement Table 1. Mean values of characteristics of audio analysis

| **Characteristic** | | **Value** |
| --- | --- | --- |
| All recordings  (N = 579) | minimum crackle entropy | 0.34 ± 0.16 |
|  | minimum crackle harmonic ratio | 0.14 ± 0.10 |
|  | minimum spectral entropy | 0.74 ± 0.12 |
|  | minimum spectral brightness 800 ratio | 0.19 ± 0.07 |
|  | median crackle duration (s) | 0.03 ± 0.01 |
|  | median crackle zero-crossing rate | 447.94 ± 154.64 |
|  | median crackle entropy | 0.56 ± 0.08 |
|  | median crackle harmonic ratio | 0.36 ± 0.09 |
|  | median squawk zero-crossing rate | 363.40 ± 159.94 |
|  | median spectral entropy | 0.80 ± 0.08 |
|  | median spectral irregularity | 0.24 ± 0.07 |
|  | maximum crackle centroid (Hz) | 756.50 ± 223.88 |
|  | maximum crackle harmonic ratio | 0.62 ± 0.15 |
|  | mean crackle frequency range (Hz) | 149.51 ± 85.77 |
|  | mean crackle entropy | 0.55 ± 0.08 |
|  | mean crackle harmonic ratio | 0.37 ± 0.08 |
|  | standard deviation spectral entropy | 0.04 ± 0.03 |
| Audio analysis (mean values per patient)  (N = 173) | minimum crackle entropy | 0.33 ± 0.13 |
|  | minimum crackle harmonic ratio | 0.12 ± 0.08 |
|  | minimum spectral entropy | 0.73 ± 0.12 |
|  | minimum spectral brightness 800 ratio | 0.20 ± 0.05 |
|  | median crackle duration (s) | 0.03 ± 0.01 |
|  | median crackle zero-crossing rate | 446.43 ± 126.20 |
|  | median crackle entropy | 0.55 ± 0.08 |
|  | median crackle harmonic ratio | 0.36 ± 0.09 |
|  | median squawk zero-crossing rate | 345.15 ± 121.22 |
|  | median spectral entropy | 0.80 ± 0.10 |
|  | median spectral irregularity | 0.24 ± 0.06 |
|  | maximum crackle centroid (Hz) | 765.00 ± 180.23 |
|  | maximum crackle harmonic ratio | 0.63 ± 0.13 |
|  | mean crackle frequency range (Hz) | 157.85 ± 65.28 |
|  | mean crackle entropy | 0.55 ± 0.07 |
|  | mean crackle harmonic ratio | 0.36 ± 0.08 |
|  | standard deviation spectral entropy | 0.04 ± 0.03 |
| For explanation of the audio analysis variables, please see text. | | |

Supplement Table 2. Factors that correlated with the length of stay in the ICU of the patients who survived

| **Factor** | **Unstandardized coefficients (B) (95% CI)** | **Standardized coefficients (r)** | **P (Value)** |
| --- | --- | --- | --- |
| Age (years) | 0.159 (-0.089 – 0.406) | 0.127 | 0.16 |
| Charlson comorbidity index | 0.409 (-1.073 – 1.891) | 0.055 | 0.59 |
| APACHE II | 0.348 (-0.117 – 0.813) | 0.148 | 0.14 |
| Baseline SOFA score | 1.443 (0.235 – 2.652) | 0.233 | **0.020** |
| Baseline hemoglobin (g/dl) | -0.437 (-1.825 – 0.950) | -0.063 | 0.53 |
| Baseline CRP (mg/dl) | -0.092 (-0.306 – 0.121) | -0.089 | 0.39 |
| Baseline PCT (ng/ml) | 0.150 (-2.082 – 2.383) | 0.014 | 0.89 |
| Baseline lung static compliance (ml/cmH_2_O) | 0.037 (-0.017 – 0.092) | 0.144 | 0.18 |
| Baseline lung resistance (cmH_2_O/L/s) | 0.025 (-0.659 – 0.709) | 0.011 | 0.94 |
| Baseline oxygenation index | 0.448 (-0.297 – 1.193) | 0.125 | 0.24 |
| Baseline ventilation equilibrium (ml/mmHg) | 0.030 (-0.020 – 0.080) | 0.124 | 0.24 |
| Auscultatory sound: normal (%) | -0.153 (-0.337 – 0.030) | -0.165 | 0.10 |
| Auscultatory sound: wheezing (%) | -0.108 (-0.594 – 0.379) | -0.044 | 0.66 |
| Auscultatory sound: rhonchi (%) | 0.170 (0.023 – 0.318) | 0.225 | **0.024** |
| Auscultatory sound: velcro (%) | 0.127 (-0.228 – 0.482) | 0.072 | 0.48 |
| Auscultatory sound: crackles (%) | 0.000 (-0.190 – 0.189) | -0.001 | 1.00 |
| Auscultatory sound: squawks (%) | 0.217 (0.065 – 0.370) | 0.275 | **0.006** |
| Auscultatory sound: tubular (%) | 0.163 (-0.020 – 0.346) | 0.176 | 0.08 |
| Auscultatory sound: diminished (%) | -0.179 (-0.314 – -0.045) | -0.258 | **0.010** |
| Auscultatory sound: absence (%) | -0.142 (-0.271 – -0.013) | -0.215 | **0.032** |
| minimum crackle entropy | -1.938 (-17.669 – 13.793) | -0.025 | 0.81 |
| minimum crackle harmonic ratio | -19.616 (-45.335 – 6.103) | -0.152 | 0.13 |
| minimum spectral entropy | -5.659 (-21.422 – 10.104) | -0.072 | 0.48 |
| minimum spectral brightness 800 ratio | -36.083 (-78.327 – 6.162) | -0.170 | 0.09 |
| median crackle duration (s) | -496.370 (-901.486 – -91.254) | -0.240 | **0.017** |
| median crackle zero-crossing rate | -0.015 (-0.031 – 0.000) | -0.192 | 0.06 |
| median crackle entropy | -21.324 (-44.067 – 1.419) | -0.186 | 0.07 |
| median crackle harmonic ratio | -11.473 (-34.712 – 11.767) | -0.099 | 0.33 |
| median squawk zero-crossing rate | -0.003 (-0.021 – 0.016) | -0.028 | 0.78 |
| median spectral entropy | -15.387 (-32.805 – 2.031) | -0.175 | 0.08 |
| median spectral irregularity | -33.141 (-70.363 – 4.081) | -0.177 | 0.08 |
| maximum crackle centroid (Hz) | -0.004 (-0.016 – 0.007) | -0.078 | 0.45 |
| maximum crackle harmonic ratio | -8.932 (-24.665 – 6.801) | -0.114 | 0.26 |
| mean crackle frequency range (Hz) | -0.041 (-0.072 – -0.010) | -0.257 | **0.010** |
| mean crackle entropy | -22.858 (-46.455 – 0.739) | -0.192 | 0.06 |
| mean crackle harmonic ratio | -16.127 (-41.557 – 9.302) | -0.127 | 0.21 |
| standard deviation spectral entropy | -68.676 (-153.349 – 15.996) | -0.161 | 0.11 |
| ICU = intensive care unit, CI = confidence intervals, APACHE II = acute physiology and chronic health evaluation II, SOFA: sequential organ failure assessment, CRP = C-reactive protein, PCT = procalcitonin | | | |

**LIST OF SUPPLEMENTARY SOUND FILES RECORDED FROM THE STUDY POPULATION**

Audio file 1: Normal lung sound

Audio file 2: Wheezing

Audio file 3: Rhonchi

Audio file 4: “Velcro” type crackles

Audio file 5: Coarse crackles

Audio file 6: Squawks

Audio file 7: Tubular sound

Audio file 8: Diminished lung sounds

Audio file 9: Absence of lung sounds

Audio file 10: Pleural rub
